# Comparative Study on Selected Properties of Modified Polyurethane Foam with Fly Ash

**DOI:** 10.3390/ijms23179725

**Published:** 2022-08-27

**Authors:** Monika Kuźnia, Beata Zygmunt-Kowalska, Artur Szajding, Anna Magiera, Rafał Stanik, Maik Gude

**Affiliations:** 1 Department of Heat Engineering and Environment Protection, Faculty of Metals Engineering and Industrial Computer Science, AGH University of Science and Technology, Mickiewicza 30 Av., 30-059 Krakow, Poland; 2 Institute of Lightweight Engineering and Polymer Technology, Technische Universität Dresden, Holbeinstraße 3, 01307 Dresden, Germany

**Keywords:** fly ash, thermal insulation materials, polyurethane composite, rigid polyurethane foam, circular economy

## Abstract

The aim of the article is to compare two types of fly ash (from the fluidized and pulverized coal combustion process) as a filler for rigid polyurethane foam. Pulverized fly ash (PFA) is widely used in building materials, while fluidized fly ash (FFA) is not currently recycled, but landfilled. The produced rigid polyurethane foams were reinforced with 5 and 10% by weight addition of fly ash from two different types of boilers. The foaming process, physical properties, morphologies and thermal degradation were subject to comparative analysis. The research indicated that fly ash intensifies the reactions of foam synthesis, most commonly, polyurethane (PU) foam with an addition of 10% PFA. What is interesting is that both ashes can be used in PU foam technology as they do not cause deterioration of the physical parameters. As shown, the addition of filler affects the morphology and impairs the brittleness. Additionally, the use of fly ash from coal combustion in the technology of polyurethane materials complies with the guidelines of the circular economy stated in the European Union legislation. Partial replacement of petrochemical components with waste filler also reduces the total energy consumption in the production of PU composites.

## 1. Introduction

Rigid polyurethane foam (RPUF) is an extremely favorable material for thermal insulation [1]. Due to the increase in energy costs and the growing awareness of society concerning the need to save energy, the demand for polyurethane is rising [2]. The main application of PU materials is the insulation of building walls. It is also used in food packaging, automotive, medical applications, aviation and furniture industries, among others [3,4,5]. The wide use of this material is due to its specific properties, which include the following: low thermal conductivity (the lowest value of all thermal insulation materials available on the market), high dimensional stability, low density, and chemical and biological resistance [6]. Moreover, the properties of the foam can be easily modified with the help of fillers, thereby producing a new composite. The characteristics of the fillers used in polyurethane composites are described below. Fossil fuels, such as hard coal, are the most popular fuels in the generation of electricity and heat in many countries around the world. Coal energy production produces a large amount of combustion by-products, such as fly ash (FA) [7]. Due to the technologies of the coal combustion process, FA is divided into two types of fly ash: fly ash from pulverized coal combustion (PCC), where the process is carried out in a high temperature rangeof 1200–1500 °C [8], and fly ash from fluidized bed combustion (FBC), where the process temperature is around 800–900 °C [9]. These two types of FA differ significantly from each other, even though they belong to the same group of waste. The chemical composition of FA is incredibly diverse and depends on the raw material (coal) and the parameters of the combustion process [10,11]. PCC fly ash is grey in color and contains silica (60–65%), alumina (25–30%), and magnetite (6–15%) [12]. This type of waste, generated during energy production, is used in the cement industry as a raw material, due to its high content of microelements, i.e., Zn, Fe, B, and Mo, as well as in agriculture, due to such macronutrients as P, K, Ca and Mg [13]. Moreover, it can be used in road construction and as a filler in mining excavations [14]. FBC fly ash is a waste that is generated more and more often and is associated with the replacement of traditional pulverized coal boilers with clean coal technology used in fluidized bed boilers [15]. This is due to the requirement to limit the emission of harmful compounds, such as SO_x_ and NO_x_ into the atmosphere [16]. FBC fly ash differs in chemical composition from PCC FA, because it contains a very high concentration of calcium and sulfur compounds (CaO and SO_3_ as CaSO_4_) [17], and, besides, it includes unburned carbon. The presence of sulfur compounds in the ash makes it more difficult to utilize than PCC fly ash. FBC FA is rarely processed and much more often landfilled [18]. The main differences between these two types of ash are their chemical composition and recyclability.

A trend in the use of fly ash in polymers can be observed. Examples of FA-reinforced polymer materials found in the scientific literature are: polyaniline (PANI) [19], geo-polymer [20], and PVC [21,22], but the most popular composite with fly ash is epoxy resin [23,24,25]. The popularity of this waste from the combustion of coal in polymeric materials is due to its low price and wide availability, thus, its neutralization is necessary because it reduces the negative impact on the environment. Few studies on the use of FA in polyurethane foams have been published. Kuźnia et al. [26] produced a polyurethane composite with the addition of FBC fly ash and performed analyses, such as Fourier-transform infrared spectroscopy (FTIR), morphology, SEM, apparent density, mechanical and thermal properties. The authors proved that the filler is evenly distributed in the matrix. The mechanical and thermal properties of the composite have shown an improvement. So far, no comparison of the properties of polyurethane foams produced with the use of FBC and PCC has been presented.

This article focuses on the comparison of using two types of fly ash to produce polyurethane composites. The effect of FA addition on the parameters of the materials was assessed on the basis of the characteristic foaming process and the conducted tests of basic parameters, such as the following: apparent density, morphology, friability, dimensional stability, water absorption and thermal properties of RPUFs. The obtained conclusions allow the differences between the composites containing the widely used PCC fly ash and the rarely reused FBC fly ash to be assessed.

## 2. Results and Discussion

### 2.1. Fly Ash Characteristics

**Figure 1:**
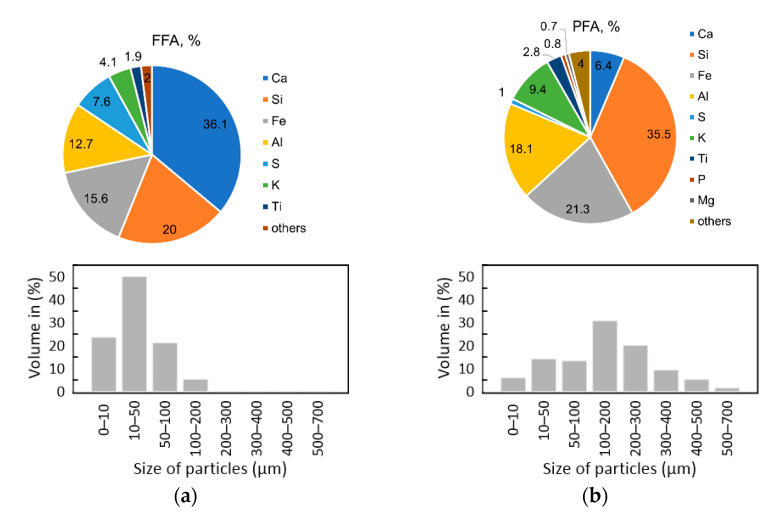
Chemical composition and particle size distribution of (**a**) FFA; (**b**) PFA.

Due to the fact that fly ash is an important part of polyurethane composites, FFA (fluidized fly ash) and PFA (pulverized fly ash) properties should be assessed before implementation of the material. XRF analysis enables the elementary composition of fly ashes to be obtained. The elemental composition of FFA and PFA ashes is shown in Figure 1a,b. The collected ash was characterized by similar densities. In the case of fly ash from fluidized combustion, the density value was 2.74 g·cm^−1^ and the density of fly ash from the pulverized combustion process was 2.15 g·cm^−1^ [27,28]. The difference between densities is most likely caused by the presence of light particles (microspheres) filled with CO_2_ and N_2_. The microspheres are present only in conventional fly ash. They do not occur in fluidized fly ash, due to the very low temperature of the coal combustion process, which is not sufficient for the formation of microspheres. The particle size distribution of the FAs is shown in Figure 1a,b.

Based on the fly ash morphology diagrams, it can be observed that FFA was characterized by a more homogeneous particle diameter distribution. The largest particles reached up to 200 µm. In the case of PFA ash, the particle size distribution was more heterogeneous: the smallest particles had diameters of several micrometers, and the largest ones could be up to 700 µm. Larger fractions found in the PFA ash may result from the presence of unburned carbon, which is characterized by large, irregular, porous particles [29].

### 2.2. Properties of Manufactured RPUF Composites

Research was carried out to determine the differences in the foaming process, physical properties, cell structure and thermal degradation of polyurethane composites containing the two types of fly ash.

#### 2.2.1. Foaming Process

**Figure 2:**
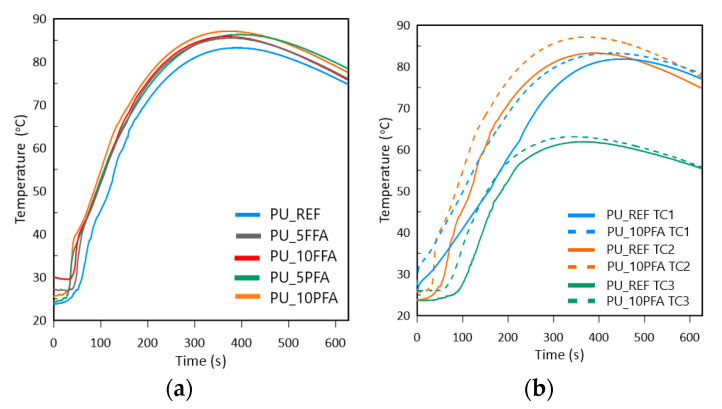
(**a**) Temperature in core foams registered by TC2 during the foaming process for all samples; (**b**) Temperature registered by TC1, TC2 and TC3 for PU_REF and PU_10PFA.

Initially, the temperature in the cup core was determined during the foaming process. Figure 2a shows the temperature distribution for each sample, as registered by the central TC2 thermocouple. Figure 2b shows temperature curves registered by TC1, TC2 and TC3 for PU_REF and PU_10PFA.

The results of measuring the maximum reaction temperatures (*T*_max_), as well as the time required (t*_T_*_max_), are presented in Table 1.

Figure 2a and Table 1 show that PU_REF had the smallest temperature rise. Both types of fillers increased the reactivity of the process. In addition, the highest temperature values were obtained for samples containing PFA: PU_5PFA and PU_10PFA. This indicated that in these samples there was a greater release of reaction heat and a higher degree of isocyanate-polyol reaction [30]. Figure 2b shows the temperature curves for modified foam (dashed line), the highest reaction temperature and temperature curves for unmodified foam (solid line), and indicating the least reactive foam. Higher temperatures were recorded for all measuring points of the foam with the added filler. The highest temperature values were recorded by the TC2 thermocouple. The lowest, on the contrary, at TC3, was the result of short direct contact of the thermocouple with the reaction mixture (thermocouple number 3 primarily registered the vapors generated during the foaming reaction) and the heat exchange with the environment.

**Figure 3:**
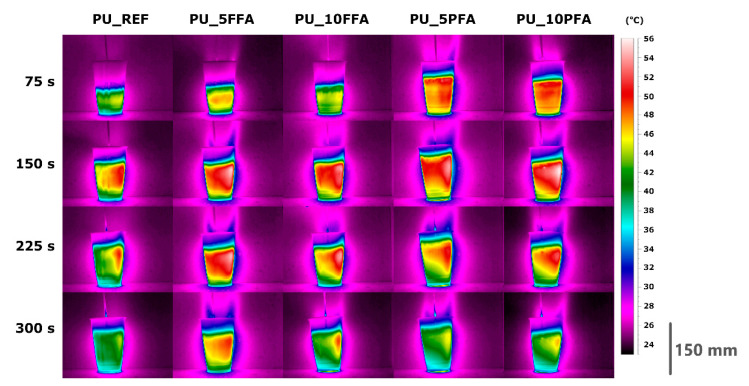
Thermo-vision photos of all samples during the RPUF growth at selected times.

As mentioned earlier, the highest temperature was obtained for PU_10PFA, at 87.1 °C, which was reached after 355 s. The remaining samples took longer to reach *T*_max_. The combination of polyols with isocyanates initiates an exothermic foaming reaction. Therefore, it was possible to study the expansion kinetics with IR (infrared radiation) [31]. Photographs were taken with a standard FLIR A615 camera equipped with a standard 25° lens. This camera is characterized by its high thermal sensitivity/NETD <0.05 °C in +30 °C/50 mK and IR resolution 640 × 480 pixels. The photos were taken at a distance of about 0.8 m from the tested object with an emissivity of 0.92. The room temperature was 22.4 °C. The maximum volume of the tested samples ranged from 360 to 400 cm^3^ depending on the type of sample. The same temperature scale was chosen for all photographs so that they could be easily compared with each other. Figure 3 shows the frames of photos taken using the IR method for various times of foam formation (after 75, 150, 225 and 300 s) for all samples.

**Figure 4:**
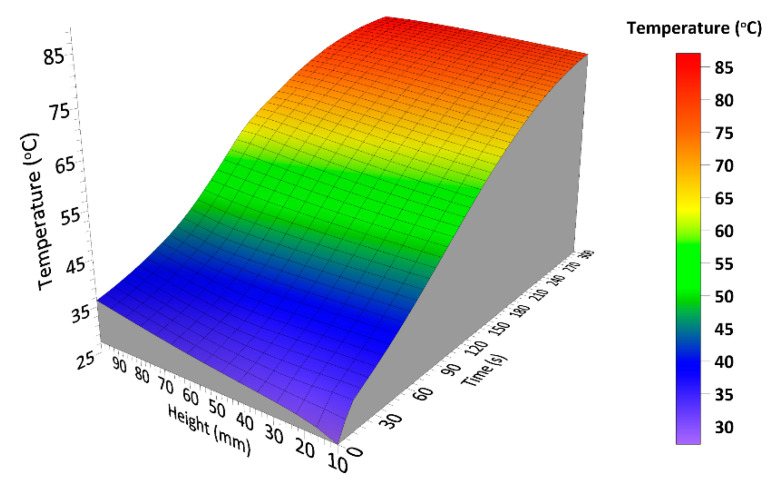
Cumulative graph for the PU_10PFA foaming parameters.

Based on the analysis of photos for 75 s, it can be observed that PU_REF grew the slowest and was the least heated, PU_5PFA and PU_10PFA showed the highest foam growth and the highest temperatures. After 150 s, the foams had already reached their maximum heights and were very hot from the accumulated heat of the reaction. Foams with fillers retain heat longer, therefore the foaming reaction takes a longer time. PU_REF foam cooled the fastest. The performed infrared expandometry confirmed the tendency of temperature distribution obtained by means of thermocouple measurements. It was observed that both types of ash significantly intensified the foaming process. The IR method is relatively new in the study of the foaming process; there are few published studies using this method to determine the effect of solid filler on the foaming process of polyurethane foam. Santiago-Calvo et al. [32] investigated the influence of graphene oxide on the process of polyurethane composite formation. The authors showed that samples with the addition of the filler were characterized by a greater reactivity of the polyurethane system. This is due to the higher viscosity of the material, which is able to trap CO_2_ causing rapid expansion. Based on the analysis of the foaming process, it was found that the best parameters were obtained for the PU_10PFA sample. Figure 4 shows a cumulative graph for this foam, the heights achieved, and temperatures, over a period of time.

The PU_10PFA sample was characterized by the maximum height and reaction temperature reached in the shortest time. It is worth mentioning that the pH of the polyol was 7.5. The pH of conventional fly ash was also 7.5, while the pH of fluidized fly ash was 12. Due to the desulphurization process that takes place in the boiler with a fluidized bed the pH increased. Calcium compounds are used as a sorbent and the presence of unused calcium oxide causes the increase of pH. Accordingly, the reaction during mixing the polyol with the fly ash does not change the temperature.

#### 2.2.2. Cell Structure

**Figure 5:**
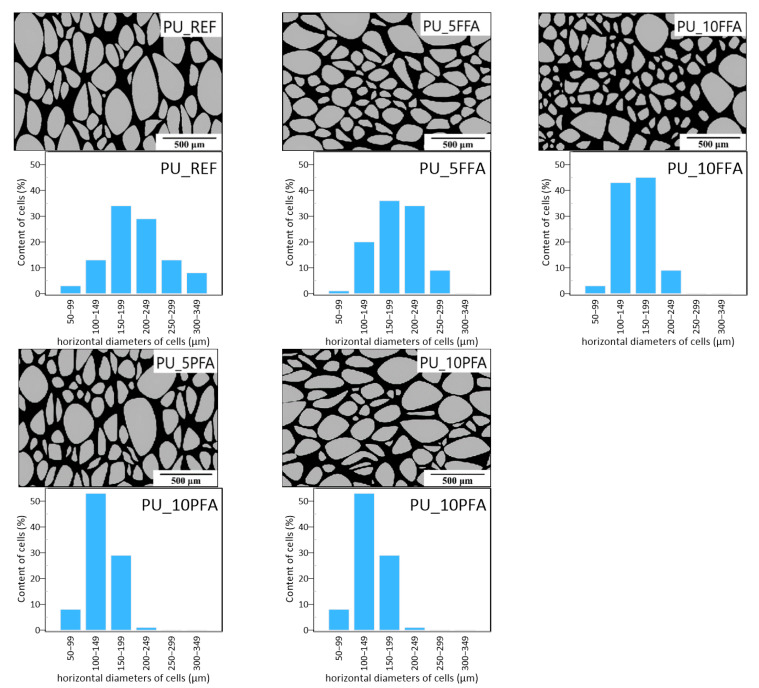
Morphologies and cell diameters of RPUFs.

The foaming process described above has a significant impact on the formation of the cellular structure. Many physical parameters, such as apparent density and thermal conductivity, depend on the morphology of the structure. Figure 5 shows pictures of the foam morphology processed in a graphic program, ImageJ. In order to determine the effect of the filler on the foam structure, cell diameters were measured and histograms with the values of horizontal diameters are shown in Figure 5.

**Figure 6:**
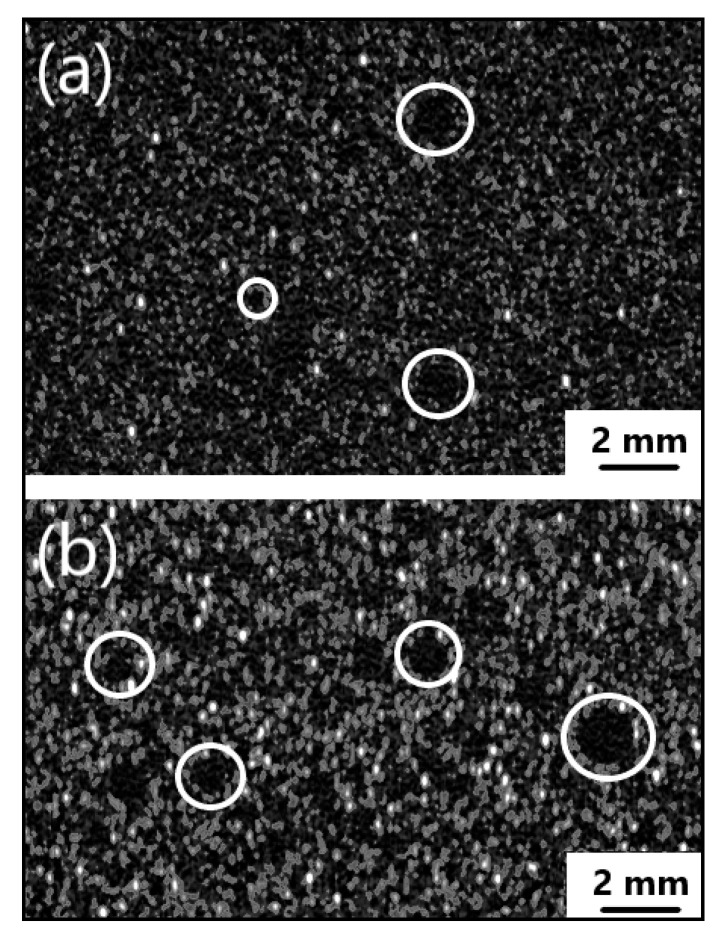
CT images (plane cross-sections) for RPUF with (**a**) 5 wt.% of filler; (**b**) 10 wt.% of filler.

The greatest variability in cell size occurred with PU_REF. Additionally, for this sample, the largest cells could be seen (horizontal diameters of 300–349 µm), which are not found in other foams. The smallest differentiation of diameters occurred in samples PU_10PFA and PU_10FFA. These foams were also characterized by the smallest cells. This signified that the average cell size of the polyurethane decreased with an increase in fly ash content. The addition of ash, like clay particles [33], acts as a pore nucleating agent and increases the viscosity of the reaction mixture and the accelerated reaction. As a consequence, many small primary cells are formed [34]. Moreover, samples containing 5% and 10% fly ash addition were scanned by computed tomography. CT images of the foams with 5% and 10% filler are shown in Figure 6.

The CT scan was used to observe the microstructure of the polyurethane composite and the effect of solid filler addition on the porosity of the material. Some examples of pore areas are marked with white circles. The porosity of the material increased as the quantity of additive increased.

#### 2.2.3. Physical Properties and Friability

Apparent density, friability, thermal conductivity (λ), changes in linear measurements (Δl) and weight loss (Δm) after 48 h under test conditions are presented in Table 2.

Density is a basic parameter which has a significant impact on the properties of the material [35].

The apparent density of the reference sample (PU_REF) was 34.8 kg·m^−3^. The addition of a filler increased the density of composites. For samples with FFA, the values of this parameter were higher than for materials with PFA. The slight differences were due to the different physical densities of the fillers. FBC fly ash has a higher density than PCC fly ash. Moreover, the increase in apparent density may result from the higher viscosity of the modified foams, which results in a limited expansion of composite cells [36]. Akkoyun et al. [37] measured the viscosity of the polyol-FA blend and showed that the viscosity increases as the filler concentration increases. The lowest friability (4.9%) was obtained for the reference foam (PU_REF). The addition of a filler increased the friability. The values of this parameter for composites with a 5% addition of fly ash (PU_5FFA, PU_5PFA) were similar. Although the type of ash had practically no effect on the numerical value. The friability of these composites was also similar to the reference foam. This meant that the 5% filler addition had no significant effect on brittleness. The addition of 10% of FA increased the friability to 7.8% (PU_10FFA) and 7.2% (PU_10PFA). Slight differences between these samples might have been caused by the presence of microspheres in the PFA fly ash composition, which are characterized by high mechanical properties resulting from their composition (a large quantity of mullite) [38]. The study of friability is the basic study of PU composites, often published in more recent literature, and its range varies from 3%, e.g., for egg shell filler [39] to approximately 20–30% for lignin particles, wood flour [40] or cork powder [41]. The dimensional stability was determined by measuring the change in the linear dimensions of the samples and, additionally, measuring the change in mass. Based on the analysis of the obtained values, it was found that the addition of both types of fillers did not affect the dimensional stability of PU foams. The values of changes in linear dimensions for all foams oscillated around 1%, and the mass changes around 0.5%. According to the applicable standards for building materials, the foam should have a dimensional stability lower than 3% (24 h, 70 °C) [42]. Consequently, all analyzed samples met the above requirements. Thermal conductivity was measured by the TPS method, widely used for thermal insulation materials [43]. The values of the thermal conductivity of the analyzed samples ranged from 30–31 mW·(m·K)^−1^. The lowest value of the coefficient was found for the PU_10PFA foam, i.e., the sample containing the highest concentration of conventional fly ash. One of the parameters influencing the value is the size of the cells [33]. The morphology of PU_10PFA was characterized by the smallest cell diameters (high viscosity of the mixture could inhibit the growth of bubbles). This correlation was not observed for the PU_10FFA sample, which may be due to the lack of microspheres in this type of ash that would enhance the insulation.

#### 2.2.4. Thermal Properties

**Figure 7:**
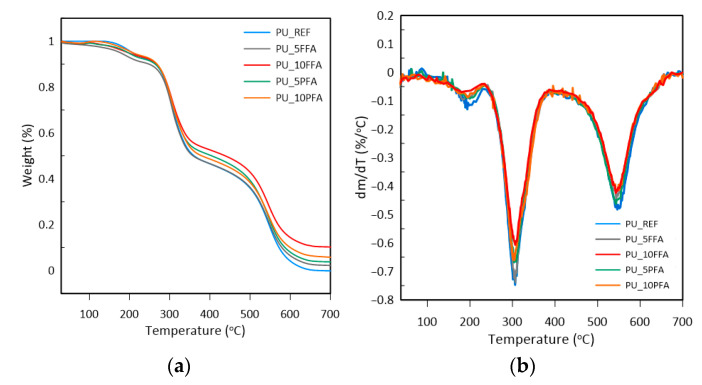
(**a**) Thermogravimetry curves; (**b**) Derivative thermogravimetry curves of RPUFs.

Based on the results presented in Table 3 and Figure 7, it was observed that the type of filler and its amount in polyurethane influenced the thermal stability of polyurethane. Table 3 shows the weight loss temperatures: 5, 10 and 50% (*T*_5%_, *T*_10%_, *T*_50%_), and the amount of residue. Due to the bimodal character of the reaction, two maxima of the mass loss rate (*T*_DTGmax1_, *T*_DTGmax2_) were determined, which are also summarized in the table.

The first noticeable weight loss on the chart (about 3%), occurred in the temperature range of approximately 110–200 °C, and was not related to the decomposition of polyurethane, but to the evaporation of moisture, unreacted isocyanate monomers and volatile substances remaining in the sample volume [44,45]. The two-stage character of polyurethane foam combustion has already been described many times in the literature. The first stage of polymer decomposition is degradation, i.e., breaking the urethane bonds. The maximum decomposition rates for this stage were in the range of 300–310 °C. In the next stage, the degradation was associated with the distribution of bonds in soft segments [46]. In the case of PU_REF, there was no charred residue. This signified that the entire sample had oxidized. In the case of the other foams, there were residues at 700 °C, and that was caused by the addition of fly ash, most of which, such as aluminosilicates, is non-flammable.

#### 2.2.5. Mechanical Properties

**Figure 8:**
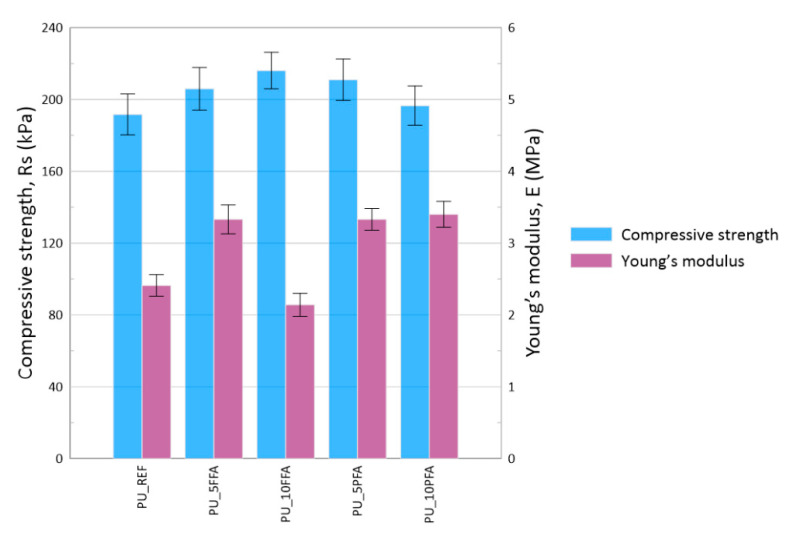
Compressive strength (Rs) and Young’s modulus, E of RPUFs.

The compressive strength and Young’s modulus of the obtained materials with different fly ash concentrations are presented in Figure 8.

The lowest compressive strength was achieved for PU_REF, Rs~190 kPa. Both types of ash improved the Rs value. The highest value of compressive strength (Rs~220 kPa) was obtained for PU_10FFA. Improvement regarding the mechanical properties may be a result of the regular distribution of the filler in the polymer and the strengthening of its matrix. As mentioned before, the addition of ash increases the apparent density of the foams, thus the increase in mechanical properties can be attributed to an increase in cross-link density [47].

#### 2.2.6. Flammability

Basic flammability tests, such as gross calorific value and limiting oxygen index (LOI), are summarized in Table 4.

The analysis of gross calorific value indicated that the addition of fly ash reduced the gross calorific value of PUR composites. The sample without the addition of fly ash was characterized by the highest value (26.4 MJ·kg^−1^). The addition of 5 and 10% of both types of fly ash caused a decrease of gross calorific value in the range of 1.3–3.0 MJ·kg^−1^ The addition of fly ash also caused a slight increase of the limited oxygen index (LOI) for composites (21.2%) compared to the unmodified PUR sample (21.0%). The decrease in the gross calorific value affected the value of the LOI.

## 3. Materials and Methods

### 3.1. Fillers Characterization

Polyurethane composites were made with two different types of fillers: fluidized fly ash (FFA) and pulverized fly ash (PFA). Both fly ash samples were obtained from one of the Polish coal power plants. This paper presents the results of ash characteristics researched for previously published articles. The description of the methodology and the results were described in detail by Kuźnia et al. [26,27,28]. The particle size of fly ash was measured by the particle size analyzer Malvern Mastersizer (Malvern Instruments, UK). This device does not have the option to display the percentage by mass, but with such unimodal distributions, the volume fraction in % is the same as the mass fraction in %. The chemical composition was analyzed by an X-ray fluorescence (XRF) spectrometer.

### 3.2. Preparation and Characterization of RPUF Samples

**Figure 9:**
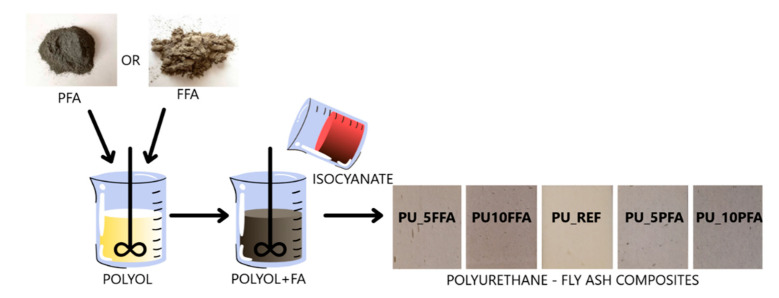
Scheme of the synthesis of polyurethane foam with fly ash (FFA, PFA).

Two types of fly ash (FFA, PFA) and a polyurethane system (containing component A and component B) were used to prepare polyurethane foam. The EKOPRODUR PM4032 system purchased from the PCC Group (BrzegDolny, Poland) was used for the production of polyurethane composites. Figure 9 shows a general scheme of the RPUF composite manufacturing process.

First, the polyol was mixed with the filler (5, 10 wt.% of FA) until a homogeneous mixture was obtained, then the isocyanate was added and mixed (1200 rpm, 8 s). In the next step, the prepared mixture was poured into a square form and the composites were left in the fume cupboard to become fully hardened (48 h). After this time, the samples were taken out of the molds, cut according to the test requirements, and analyzed. Additionally, for analysis of the foaming process, PUR foams were also produced in plastic cups. As part of the research, two foams with the addition of 5% and 10% fluidized fly ash (PU_5FFA, PU_10FFA), two foams with the addition of 5% and 10% conventional fly ash (PU_5PFA, PU_10PFA) and a reference sample without filler (PU_REF) were assembled.

**Figure 10:**
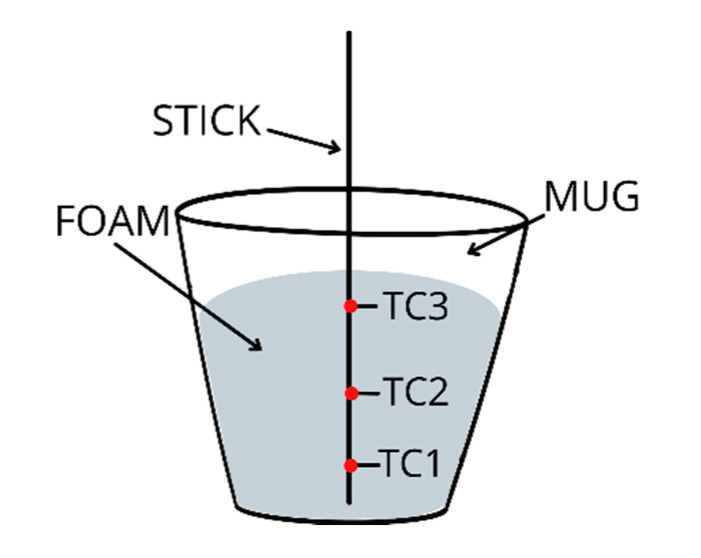
Scheme of measuring temperature distribution during foam growth.

The characteristics of the foaming process and the influence of fly ash on the foam growth process were analyzed by measuring the temperature. The thermocouple distribution can be seen in Figure 10.

The temperature was measured by using three thermocouples located on the axis of the plastic cup. Thermocouples were placed on the wooden stick at distances from the ground: 1 cm thermocouple 1 (TC1), 5 cm thermocouple 2 (TC2), 9 cm thermocouple 3 (TC3). The temperature distribution on the surface during the foaming process was also measured. The study used the FLIR A600 camera and FLIR Systems AB.

The surface morphology and the influence of the filler on the composite cell structure were evaluated using a Keyence VHX-900F stereoscopic microscope (Keyence, Osaka, Japan). ImageJ software (version 1.53k, Wayne Rasband, Kenigston, MD, USA) was used to determine the dimensions of the diameters of the polyurethane cells. The photographs were processed graphically and the cell surfaces were marked in grey and the polymer matrix in black.

To observe the effect of the filler on the foam morphology, it was necessary to perform a 3D CT scan (computed tomography). High resolution computed tomography, the Phoenix V|tome|x L450 (Waygate Technologies, Huerth, Germany), was used to analyze the distribution of the filler in the foam.

To determine the apparent density, the method of volume and mass measurement was calculated in accordance with the ASTM D1622-03 standard. It consisted of measuring the dimensions of the sample, calculating the volume, and then weighing and calculating the density from the mass to volume ratio. The thermal conductivity coefficients were obtained in accordance with ISO 2207-2 using the Hot Disk TPS 3500 Thermal Constants Analyzer (Hot Disk, Gothenburg, Sweden) at room temperature (25 °C).

The friability was evaluated during the test carried out according to the ASTM C421-08 standard. The previously weighed samples were placed in a box with oak cubes and rotated in accordance with the parameters specified in the standard. After the process the foams were re-weighed.

Dimensional stability was designated in accordance with the ASTM D2126-09 standard. The length of samples was measured and foams were weighed before being placed in the oven and then removed at the end of the process. PU composites were exposed for 48 h at a temperature of 70 °C.

Thermogravimetric analysis (TGA) of the manufactured composites was performed on the Mettler Toledo TGA/DSC 3+ (Mettler Toledo, Greifensee, Switzerland) to measure the thermal properties of the foams produced. Samples weighing 10 mg were placed in ceramic pans and analyzed. Tests were carried out under the following conditions: air, 25–700 °C and heating rate 10 °C min^−1^. Specified temperatures of weight loss of 5% (*T*_5%_), 10% (*T*_10%_), 50% (*T*_50%_) and the residue at 700 °C were determined.

Mechanical properties of the foams were assessed in accordance with EN 826:2013 using the universal testing machine, Zwick 1435, at 10% relative deformation (σ_10_). Compressive strength (Rs) and Young’s modulus (E), were calculated from the obtained stress-strain curves.

In order to check the reaction to fire, the gross calorific value was calculated in accordance with the ISO 1716:2018 standard using the LECO AC500 isoperibolic calorimeter (LECO Corporation, Saint Joseph, MI, USA).

The limiting oxygen index (LOI) was measured according to ISO 4589-2: 2017. The samples used for the test were 150 × 10 × 10 mm^3^.

## 4. Conclusions

Both types of ash used as fillers (pulverized fly ash, fluidized fly ash) intensified the foaming process, but the reaction was the most intense for PUR with the addition of 10% pulverized fly ash. For this sample, the reaction temperature was the highest and was reached in the shortest time and recorded as 87.1 °C after 355 s. The remaining samples took longer to reach *T*_max_. The lowest temperature of the foaming process, i.e., 83.3 °C, was characterized by the reference PUR sample.

The addition of the filler significantly affected the morphology of the composite. This study showed that the diameters of the foam cells decreased with an increase in the concentration of the filler in the product. The largest cells were characteristic of the reference PUR sample, where the dimensions of the diameters of the cells wer up to 350 µm. The addition of a filler increased the porosity of the material.

The addition of both fillers increased the friability of the composite. The highest values of this parameter were achieved for the PUR with the addition of 10% fluidized and pulverized fly ash. The friability for these samples was 7.8 and 7.2%, respectively. The analysis of the physical properties indicated that both ashes can be used in the foam because they do not cause deterioration of these parameters.

Fly ash fillers have improved the thermal properties of polyurethane composites, which is related to the barrier effect of the filler and prevention of the release of gases from foam cells. The reference PUR sample was completely oxidized in an oxygen atmosphere. For samples containing fly ash, the residue is from 3 to 10% depending on the type and amount of ash. The presence of residues results from fly ash components, most of which, such as aluminosilicates, are non-flammable.

The addition of both types of fly ash improved the mechanical properties of the composites with the highest compressive strength parameters of approximately 220 kPa obtained for the sample containing the 10% addition of fluidized fly ash.

Concluding, ash from fluidized and conventional combustion processes can be a good modifier for rigid polyurethane foams. Due to the valuable properties of the microspheres present in pulverized fly ash, it is worth considering the possibility of mixing polyurethane foams with fluidized fly ash and using the obtained “mixed filler” for the production of new polyurethane composites.

The use of fly ash as a filler in the production of polymer composites ensures that sustainable materials are obtained. In the near future, all materials produced will have to contain a waste additive, which is one of the guidelines for achieving climate neutrality in the European Union.

## Figures and Tables

**Table 1:** Characteristic parameters of the foaming process.

Sample	*T*_max_ (°C)	t*_T_*_max_ (s)
PU_REF	83.3	379
PU_5FFA	85.6	389
PU_10FFA	85.9	360
PU_5PFA	86.4	389
PU_10PFA	87.1	355

**Table 2:** Physical properties and friability of the RPUF samples.

Sample	Apparent Density (kg·m^−3^)	Friability (%)	Δl [48 h, 70 °C] (%)	Δm [48 h, 70 °C] (%)	λ (mW·(m·K)^−1^)
PU_REF	34.8 ± 0.5	4.9 ± 0.4	0.88 ± 0.09	0.52 ± 0.07	31.1 ± 0.4
PU_5FFA	38.0± 0.5	5.8 ± 0.8	0.99 ± 0.01	0.58 ± 0.05	30.3 ± 0.7
PU_10FFA	40.4 ± 0.8	7.8 ± 0.9	0.93 ± 0.09	0.57 ± 0.06	30.6 ± 0.9
PU_5PFA	37.7 ± 0.6	5.7 ± 0.9	1.01 ± 0.09	0.62 ± 0.01	30.8 ± 0.8
PU_10PFA	39.9 ± 0.9	7.2 ± 0.8	1.03 ± 0.05	0.60 ± 0.04	30.1 ± 0.4

**Table 3:** Thermal degradation parameters of RPUF composites.

Sample	*T*_5%_ (°C)	*T*_10%_ (°C)	*T*_50%_ (°C)	*T*_DTGmax1%_ (°C)	*T*_DTGmax2%_ (°C)	Residue at 700 °C (%)
PU_REF	271	286	417	310	557	0.00
PU_5FFA	264	284	439	307	549	2.92
PU_10FFA	268	286	488	306	543	10.27
PU_5PFA	265	285	464	309	542	3.79
PU_10PFA	270	286	450	300	551	5.83

**Table 4:** Flammability properties of RPUFs.

Sample	Gross Calorific Value (MJ·kg^−1^)	LOI (%)
PU_REF	26.4	21.0
PU_5FFA	25.1	21.0
PU_10FFA	23.6	21.2
PU_5PFA	24.6	21.2
PU_10PFA	23.4	21.2

## Data Availability

Not applicable.
